# A synergistic effect of variability in estimated glomerular filtration rate with chronic kidney disease on all-cause mortality prediction in patients with type 2 diabetes: a retrospective cohort study

**DOI:** 10.1186/s12933-021-01399-z

**Published:** 2021-10-18

**Authors:** Yu-Shan Chang, Yu-Hsuan Li, I-Te Lee

**Affiliations:** 1 Division of Endocrinology and Metabolism, Department of Internal Medicine, Taichung Veterans General Hospital, No. 1650, Section 4, Taiwan Boulevard, Taichung, 40705 Taiwan; 2 School of Medicine, Chung Shan Medical University, Taichung City, 40201 Taiwan; 3 Department of Computer Science and Information Engineering, National Taiwan University, Taipei, 10617 Taiwan; 4 School of Medicine, National Yang Ming Chiao Tung University, Taipei, 11221 Taiwan

**Keywords:** Annual, Chronic kidney disease, Estimated glomerular filtration, Mortality, Standard deviation, Type 2 diabetes, Variability

## Abstract

**Background:**

The combination of diabetes mellitus (DM) and chronic kidney disease (CKD) is associated with a high risk of mortality. Annual assessment of the estimated glomerular filtration rate (eGFR) is recommended for patients with DM. We investigated the effect of variability in annual eGFR values on all-cause mortality in patients with type 2 DM.

**Methods:**

In this retrospective cohort study, we enrolled patients with eGFR data between 01 Aug 2017 and 31 July 2018. We defined the index eGFR as the first available eGFR value within the enrollment year and collected additional annual eGFR data from the previous three years. A total of 3592 patients with type 2 DM were enrolled, including 959 patients with CKD (index eGFR < 60 mL/min/1.73 m^2^) and 2633 patients without CKD. We assessed eGFR variability by using the standard deviation (SD) of the three annual eGFR and index eGFR values. We divided patients into subgroups according to the median SD of their annual eGFR (7.62 mL/min/1.73 m^2^). The primary endpoint was all-cause mortality after the index eGFR was assessed.

**Results:**

During a median follow-up of 19 months (interquartile range: 18‒20 months), 127 (3.5%) deaths occurred among all 3592 enrolled patients. The highest mortality risk was observed in the high SD with CKD group, with a hazard ratio (HR) of 2.382 [95% confidence interval (CI) 1.346‒4.215] in comparison to the low SD without CKD group after adjusting for the associated factors. In patients without CKD, a high SD was an independent risk factor for mortality (HR = 2.105, 95% CI 1.256‒3.528). According to the C-index, the mortality prediction ability was better for the index eGFR + SD model than for the index eGFR alone model (0.671 vs. 0.629, P < 0.001).

**Conclusion:**

There was a synergistic effect of eGFR variability with single-measured eGFR for the prediction of mortality in patients with type 2 DM. The SD of the annual eGFR values was also an independent predictor of mortality in patients with an eGFR > 60 mL/min/1.73 m^2^.

**Supplementary Information:**

The online version contains supplementary material available at 10.1186/s12933-021-01399-z.

## Background

Chronic kidney disease (CKD) is a critical risk factor for death [1]. According to the systematic analysis of the Global Burden of Disease Study, approximately 1.23 million people died from CKD in 2017, and the global all-age mortality rate for CKD increased by 41.5% between 1990 and 2017 [2]. The global prevalence of CKD between stages 3 and 5 was reported to be approximately 10.6%, and diabetes mellitus (DM) is a major risk factor for CKD [3]. As the number of people with DM has been increasing worldwide [4], DM has become the most important cause of new-onset CKD [5, 6]. Type 2 DM, the major type of DM, was responsible for more than 400 thousand deaths of CKD and was reported to be the second leading cause of CKD-related death in 2019 [6].

A reduction in estimated glomerular filtration rate (eGFR), as well as the presence of albuminuria, is an independent predictor for mortality in patients with type 2 DM [7–9]. Annual assessments of eGFR and urinary albumin-to-creatinine ratio (UACR) are recommended in the clinical management of patients with DM [10–12]. In particular, the stage of CKD is recommended to be defined using the eGFR value [13, 14]. However, a meta-analysis reported that mortality risk was not linearly correlated with eGFR in individuals with an eGFR ≥ 60 mL/min/1.73 m^2^ [15]. An eGFR higher than the normal range might be associated with an increased risk of mortality in patients with type 2 DM [16]. Therefore, it is not sufficient to predict mortality using a single-measurement of eGFR in patients with eGFR ≥ 60 mL/min/1.73 m^2^, and other associated risk factors for mortality should be assessed in patients with type 2 DM without hypofiltration [17].

Although the rate of annual eGFR assessments could be greater than 80%, the rate of annual UACR assessments was less than 50% in patients with type 2 DM [18]. Similarly, based on the data of National Health Insurance in Taiwan, the rate of annual eGFR assessments was 74.2% and that of UACR was 35.9% in 2014 [19]. The variability in eGFR has been reported to be associated with new-onset DM in a Korean population and with CKD progression in the population with DM [20, 21]. Perkins et al. [22] reported that eGFR variability could significantly predict mortality in patients with stage 3 CKD. However, a recent post hoc analysis from the ADVANCE trial reported that eGFR variability was significantly associated with the primary outcome, including major macrovascular events, new or worsening nephropathy and all-cause mortality, but eGFR variability was not significantly associated with all-cause mortality as a secondary outcome in patients with type 2 DM [23]. These inconsistent results might be explained by the various CKD stages and presence of DM in the study population. We hypothesized the existence of a synergistic effect of eGFR and previous eGFR variability on mortality prediction in patients with type 2 DM. Therefore, we conducted a retrospective cohort study to investigate the association between the standard deviation (SD) of previously measured eGFR values and mortality in type 2 DM patients with and without CKD.

## Methods

### Study design and population

This observational cohort study was conducted in Taichung Veterans General Hospital. We retrospectively screened candidates from outpatients who had visited the Division of Endocrinology and Metabolism. The inclusion criteria were: (1) adults with type 2 DM; and (2) at least one serum creatinine level assessed between 01 Aug 2017 and 31 July 2018. We defined the index eGFR as the eGFR calculated using the first available serum creatinine level within the enrollment year. We further collected additional eGFR data from the past three years, and recorded the first-available eGFR during the annual period. We excluded patients who met any of the following conditions: (1) pregnancy, (2) the index eGFR assessed during hospitalization or in the emergency room, (3) end-stage renal disease when the index eGFR was assessed, and (4) incomplete annual eGFR data within 1 and 3 years prior to the index eGFR.

After the index eGFR was assessed, the occurrence of all-cause mortality was recorded. Information on deaths registered through August 31, 2019, was obtained from the Ministry of Health and Welfare, Executive Yuan, Taiwan. This research protocol was approved by the Institutional Review Board of Taichung Veterans General Hospital, and the need for informed consent was waived due to the retrospective cohort study design.

### Assessments of risk factors

The laboratory data of hemoglobin A1c (HbA1c), total cholesterol, high-density lipoprotein (HDL) cholesterol, triglycerides, and creatinine were recorded in the enrollment year. HbA1c was measured using cation-exchange high-performance liquid chromatography (NGSP certified; G8, TOSOH, Tokyo, Japan). Serum lipid profiles and creatinine levels were measured by commercial kits (Beckman Coulter, Fullerton, CA, USA). The eGFR was calculated using the formula: eGFR = 186 × [serum creatinine (mg/dL)]^−1.154^ × [age (years)]^−0.203^ (× 0.742 if female) based on the Modification of Diet in Renal Disease (MDRD) equation [13]. CKD was defined as an index eGFR < 60 mL/min/1.73 m^2^. The mean eGFR was calculated as the average of the index eGFR and other three annual eGFR values recorded within the three years prior to the index eGFR. The variability in annual eGFR was calculated using the SD of the four eGFR datasets mentioned above. Patients were divided into the high and low SD of annual eGFR groups based on the median of 7.62 mL/min/1.73 m^2^. Hypertension was defined as a systolic blood pressure ≥ 140 mmHg, a diastolic blood pressure ≥ 90 mmHg, or the current use of an antihypertensive drug. Obesity in Taiwan was defined as a body mass index (BMI) ≥ 27 kg/m^2^ [24, 25]. Low HDL cholesterol was defined as an HDL cholesterol level < 50 mg/dL (1.29 mmol/L) in women or < 40 mg/dL (1.03 mmol/L) in men. The UACR was calculated using the ratio of urine albumin (mg/dL) to urine creatinine (g/dL), and albuminuria was defined as a UACR ≥ 300 mg/g.

### Statistical analysis

Continuous data are presented as the mean ± standard deviation; differences among the four study groups were analyzed using one-way analysis of variance, and the Scheffé test was conducted for the post hoc analysis of the differences between the paired groups. Categorical data are summarized as numbers with percentages (%) and were compared among groups using the chi-square test. The primary endpoint was all-cause mortality.

The cumulative risk of all-cause mortality was assessed using Kaplan–Meier analysis; the log-rank test was used to determine whether the survival rates among groups were significantly different. Multivariable Cox proportional hazards regression analysis was conducted to identify the independent predictors of mortality; hazard ratio (HR) and 95% confidence interval (CI) were calculated.

We compared the mortality prediction of the different models by examining the C-index. The performance of the index eGFR + SD model was compared with that of the index eGFR alone model using the integrated discrimination improvement (IDI) and continuous net reclassification improvement (NRI) to quantify the improvement in predictive ability. The relationship between the annual eGFR values and time was determined by Spearman’s correlation. A two-sided P value < 0.05 was considered statistically significant. Statistical analysis was performed using SPSS v22.0 (IBM Corp., Armonk, NY, USA) and R software v3.4.

## Results

**Fig. 1:**
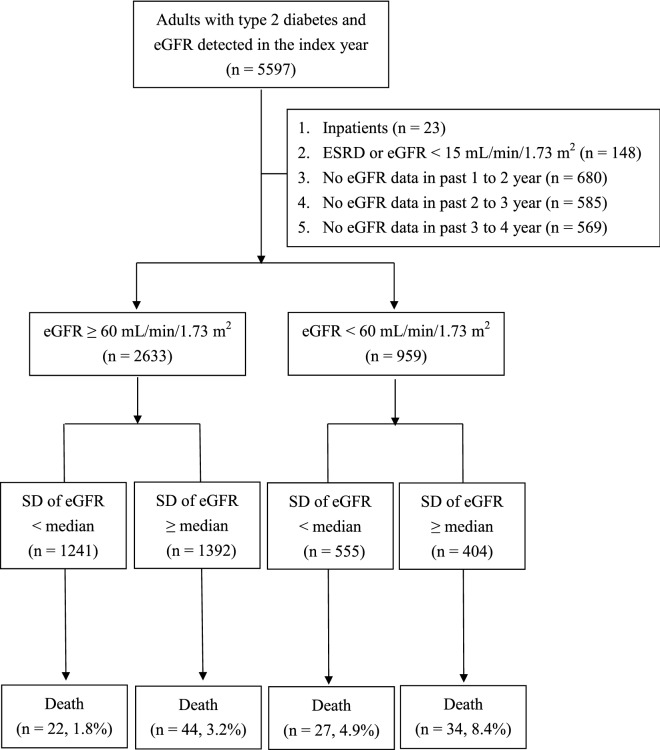
Flow diagram of the enrollment of study subjects. *ESRD* end-stage renal disease, *eGFR* estimated glomerular filtration rate, *SD* standard deviation

Of the 5597 patients with type 2 DM, 3592 patients who met the study criteria were enrolled, including 2633 patients without CKD and 959 patients with CKD. Furthermore, we divided all patients into four subgroups based on the median SD of their annual eGFR. There were 1241 patients in the low SD without CKD group, 1392 patients in the high SD without CKD group, 555 patients in the low SD with CKD group, and 404 patients in the high SD with CKD group (Fig. 1).

The clinical characteristics of the enrolled patients are shown in Table 1. Among the four subgroups, there were significant differences in age (P < 0.001), BMI (P = 0.002), duration of DM (P < 0.001), systolic blood pressure (P < 0.001), HbA1c (P = 0.005), total cholesterol (P < 0.001), HDL cholesterol (P < 0.001), triglycerides (P < 0.001), index eGFR (P < 0.001), mean eGFR (P < 0.001), SD of annual eGFR (P < 0.001), and proportions of males (P < 0.001), current smokers (P < 0.001), individuals with cardiovascular disease (CVD) history (P < 0.001), individuals with hypertension (P < 0.001), individuals with albuminuria (P < 0.001), individuals using angiotensin-converting enzyme (ACE) inhibitors or angiotensin II receptor antagonists (ARBs) (P < 0.001), individuals using antiplatelet drugs (P < 0.001), individuals using insulin therapy (P < 0.001), and individuals using oral antidiabetic drugs (P = 0.008).

**Table 1:** Characteristics of the enrolled patients categorized based on CKD and SD of eGFR

	CKD (−) n = 2633		CKD (+) N = 959		P
	Low SD (n = 1241)	High SD (n = 1392)	Low SD (n = 555)	High SD (n = 404)	
Age (year)	65.0 ± 9.8	63.2 ± 9.6^a^	73.0 ± 10.0^ab^	71.3 ± 10.5^ab^	< 0.001
Male, n (%)	773 (62.3%)	629 (45.2%)^a^	339 (61.1%)^b^	208 (51.5%)^abc^	< 0.001
Current smoking, n (%)	142 (11.4%)	127 (9.1%)	32 (5.8%)^ab^	25 (6.2%)^a^	< 0.001
CVD history, n (%)	155 (12.5%)	198 (14.2%)	128 (23.1%)^ab^	114 (28.2%)^ab^	< 0.001
BMI (kg/m^2^)	25.7 ± 3.9	25.8 ± 4.4	25.8 ± 4.0	26.6 ± 4.2^ab^	0.002
Duration of diabetes (year)	13.2 ± 7.6	12.4 ± 7.1	17.2 ± 8.6^ab^	16.0 ± 8.8^ab^	< 0.001
Hypertension, n (%)	947 (76.3%)	1033 (74.2%)	521 (93.9%)^ab^	382 (94.6%)^ab^	< 0.001
Systolic BP (mmHg)	135 ± 18	135 ± 18	141 ± 19^ab^	142 ± 22^ab^	< 0.001
Diastolic BP (mmHg)	77 ± 10	76 ± 11	76 ± 11	76 ± 13	0.231
HbA1c (%)	7.3 ± 1.3	7.5 ± 1.4	7.4 ± 1.4	7.6 ± 1.7^*^	0.005
Total cholesterol (mmol/L)	4.0 ± 0.8	4.1 ± 0.9^a^	4.0 ± 0.8^b^	4.0 ± 0.9^b^	< 0.001
HDL cholesterol (mmol/L)	1.3 ± 0.4	1.3 ± 0.4	1.3 ± 0.4^b^	1.2 ± 0.4^ab^	< 0.001
Triglycerides (mmol/L)	1.4 ± 0.8	1.5 ± 1.3^a^	1.6 ± 1.3^a^	1.9 ± 2.0^abc^	< 0.001
Index eGFR (mL/min/1.73 m^2^)	84.6 ± 16.1	93.4 ± 21.1^a^	44.2 ± 11.4^ab^	42.9 ± 12.3^ab^	< 0.001
Mean of eGFR (mL/min/1.73 m^2^)	85.2 ± 15.9	97.8 ± 20.1^a^	46.7 ± 11.3^ab^	54.7 ± 15.0^*#†^	< 0.001
SD of eGFR (mL/min/1.73 m^2^)	5.0 ± 1.6	13.7 ± 7.1^a^	4.4 ± 1.7^b^	13.6 ± 7.2^*†^	< 0.001
Albuminuria	69 (5.6%)	87 (6.3%)	143 (25.8%)^ab^	117 (29.0%)^ab^	< 0.001
ACE inhibitor or ARB, n (%)	491 (39.6%)	489 (35.1%)	312 (56.2%)^ab^	223 (55.2%)^ab^	< 0.001
Antiplatelet, n (%)	339 (27.3%)	371 (26.7%)	243 (43.8%)^ab^	198 (49.0%)^ab^	< 0.001
Statins, n (%)	929 (74.9%)	1027 (73.8%)	414 (74.6%)	291 (72.0%)	0.701
Insulin therapy, n (%)	244 (19.7%)	321 (23.1%)^a^	170 (30.6%)^ab^	144 (35.6%)^ab^	< 0.001
Oral antidiabetic drugs	1124 (90.6%)	1255 (90.2%)	479 (86.3%)	349 (86.4%)	0.008
Insulin secretagogues, n (%)	484 (39.0%)	519 (37.3%)	227 (40.9%)	156 (38.6%)	0.505
Metformin, n (%)	508 (40.9%)	559 (40.2%)	125 (22.5%)^ab^	115 (28.5%)^abc^	< 0.001
Thiazolidinediones, n (%)	298 (24.0%)	359 (25.8%)	143 (25.8%)	99 (24.5%)	0.721
DPP4 inhibitors, n (%)	748 (60.3%)	816 (58.6%)	349 (62.9%)	253 (62.6%)	0.251
SGLT2 inhibitors, n (%)	132 (10.6%)	203 (14.6%)^a^	28 (5.0%)^ab^	22 (5.4%)^ab^	< 0.001
Mortality, n (%)	22 (1.8%)	44 (3.2%)^a^	27 (4.9%)^a^	34 (8.4%)^abc^	< 0.001
Incidence of mortality (deaths/100 person-years)^d^	1.1	2.0	3.2	5.6	< 0.001

Continuous data are presented as the mean ± SD, and categorical data are presented as numbers (%)

CKD was defined as an index eGFR < 60 mL/min/1.73 m^2^, and SD of eGFR was grouped based on the median of 7.62 mL/min/1.73 m^2^

*ACE* angiotensin-converting enzyme, *ARB* angiotensin II receptor antagonist, *BMI* body mass index, *BP* blood pressure, *CVD* cardiovascular disease, *DPP4* dipeptidyl peptidase-4, *eGFR* estimated glomerular filtration rate, *HbA1c* hemoglobin A1c, *HDL* high-density lipoprotein, *SD* standard deviation, *SGLT2* sodium glucose cotransporter 2

^a^^–c^Indicates statistically significant differences (P < 0.05) compared to the low SD without CKD, high SD without CKD, and low SD with CKD groups, respectively; only one of the three markers, in the order of priority, is used between the compared groups

^d^Using log rank test

Using the post hoc analyses, patients with low SD had a significantly older age (65.0 ± 9.8 vs. 63.2 ± 9.6 years, P < 0.001), a higher proportion of males (62.3 vs. 45.2%, P < 0.001), lower total cholesterol (4.0 ± 0.8 vs. 4.1 ± 0.9 mmol/L, P < 0.001), lower triglycerides (1.4 ± 0.8 vs. 1.5 ± 1.3, P = 0.013), a lower index eGFR (84.6 ± 16.1 vs. 93.4 ± 21.1 mL/min/1.73 m^2^, P < 0.001), a lower mean eGFR (85.2 ± 15.9 vs. 97.8 ± 20.1 mL/min/1.73 m^2^, P < 0.001), a lower SD of annual eGFR (5.0 ± 1.6 vs. 13.7 ± 7.1 mL/min/1.73 m^2^, P < 0.001), and lower proportions of insulin use (19.7 vs. 23.1%, P = 0.038) and sodium glucose cotransporter 2 (SGLT2) inhibitors use (10.6 vs. 14.6%, P = 0.003) in the group of patients without CKD. However, in the group of patients with CKD, the subgroup with a low SD had a significantly higher proportion of males (61.1 vs. 51.5%, P = 0.003), lower triglycerides (1.6 ± 1.3 vs. 1.9 ± 2.0, P = 0.007), a lower mean eGFR (46.7 ± 11.3 vs. 54.7 ± 15.0 mL/min/1.73 m^2^, P < 0.001), a lower SD of annual eGFR (4.4 ± 1.7 vs. 13.6 ± 7.2 mL/min/1.73 m^2^, P < 0.001), and a lower proportion of metformin use (22.5 vs. 28.5%, P = 0.043) (Table 1).

**Fig. 2:**
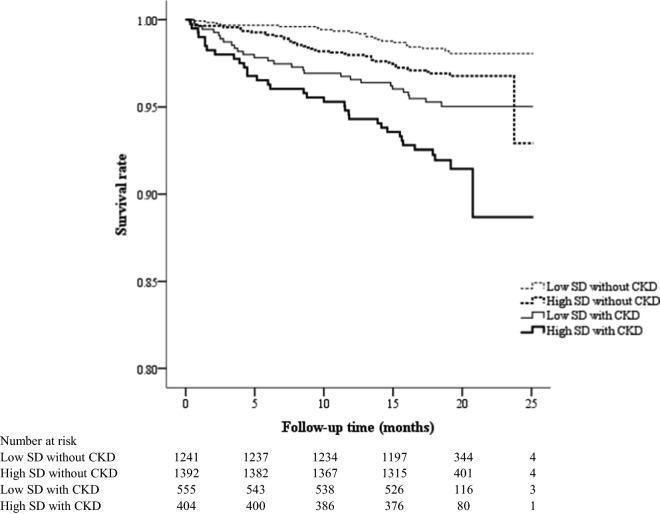
Kaplan–Meier curves showing the survival rates across the four groups defined based on chronic kidney disease (CKD) and standard deviation (SD) of annual estimated glomerular filtration rate (log-rank test P < 0.001)

During a median follow-up of 19 months (interquartile range: 18‒20 months), a total of 127 (3.5%) deaths occurred among all 3592 enrolled patients. The baseline characteristics and mortality cause of expired patients are shown in the Additional file 1: Table S1. The annual incidence of mortality was 1.1 per 100 person-years in the low SD without CKD group, 2.0 per 100 person-years in the high SD without CKD group, 3.2 per 100 person-years in the low SD with CKD group, and 5.6 per 100 person-years in the high SD with CKD group. Based on the Kaplan–Meier analysis, the survival rates were significantly different among these four groups (log-rank test P < 0.001, Fig. 2). The mortality risks were still significantly different among these four groups (P = 0.003) after adjusting for the associated risk factors selected from Table 1 by using multivariate Cox regression analysis. Among the patients without CKD, a high SD of annual eGFR significantly predicted mortality with an HR of 2.105 (95% CI 1.256‒3.582); among the patients with a low SD of annual eGFR, however, CKD did not significantly predict mortality (HR = 1.224, 95% CI 0.675‒2.217). There was a synergistic effect of CKD and a high SD of annual eGFR on mortality prediction, with an HR of 2.382 (95% CI 1.346‒4.215) after adjusting for age, sex, current smoking status, CVD history, obesity, duration of DM, hypertension, HbA1c, total cholesterol, HDL cholesterol, triglycerides, albuminuria, the use of ACE inhibitors or ARBs, and the use of statins, antiplatelet agents, insulin, metformin, and SGLT2 inhibitors (Table 2).

**Table 2:** Cox proportional hazard regression models for the association between the risk factors and mortality

	HR	95% CI	P	HR	95% CI	P	HR	95% CI	P	HR	95% CI	P
Low SD without CKD group			< 0.001			< 0.001			< 0.001			0.002
High SD without CKD group	2.123	(1.268, 3.555)	0.004	2.096	(1.251, 3.511)	0.005	2.113	(1.261, 3.542)	0.005	2.105	(1.256, 3.528)	0.005
Low SD with CKD group	2.066	(1.169, 3.649)	0.012	1.774	(0.999, 3.149)	0.050	1.502	(0.841, 2.682)	0.169	1.224	(0.675, 2.217)	0.505
High SD with CKD group	4.091	(2.380, 7.029)	< 0.001	3.497	(2.019, 6.057)	< 0.001	2.966	(1.704, 5.163)	< 0.001	2.382	(1.346, 4.215)	0.003
Age ≥ 65 years	3.261	(2.097, 5.070)	< 0.001	2.767	(1.761, 4.348)	< 0.001	2.654	(1.684, 4.183)	< 0.001	2.631	(1.671, 4.144)	< 0.001
Male	1.692	(1.175, 2.436)	0.005	1.636	(1.122, 2.385)	0.011	1.498	(1.025, 2.189)	0.037	1.496	(1.016, 2.203)	0.041
Current smoker				0.987	(0.507, 1.922)	0.970	1.076	(0.550, 2.107)	0.830	1.103	(0.562, 2.164)	0.775
CVD history				1.293	(0.867, 1.927)	0.208	1.281	(0.827, 1.986)	0.267	1.287	(0.833, 1.987)	0.256
Obesity^a^				0.992	(0.681, 1.445)	0.967	1.006	(0.689, 1.467)	0.977	0.993	(0.680, 1.450)	0.973
Duration of diabetes ≥ 13 years^b^				1.594	(1.067, 2.380)	0.023	1.455	(0.967, 2.189)	0.072	1.496	(0.992, 2.256)	0.055
Hypertension				1.898	(0.940, 3.834)	0.074	1.681	(0.809, 3.494)	0.164	1.507	(0.720, 3.154)	0.277
Current use of ACE inhibitors or ARBs							1.220	(0.839, 1.773)	0.297	1.218	(0.839, 1.769)	0.300
Current use of statins							0.517	(0.361, 0.741)	< 0.001	0.524	(0.364, 0.755)	< 0.001
Current use of antiplatelet agents							1.155	(0.774, 1.724)	0.482	1.107	(0.742, 1.650)	0.618
Current use of insulin							1.454	(0.999, 2.116)	0.050	1.295	(0.865, 1.939)	0.210
Current use of metformin							0.690	(0.449, 1.061)	0.091	0.705	(0.458, 1.085)	0.112
Current use of SGLT2 inhibitors							0.159	(0.039, 0.649)	0.010	0.184	(0.045, 0.754)	0.019
HbA1c ≥ 7%										0.798	(0.551, 1.154)	0.231
Total cholesterol ≥ 4.14 mmol/L										0.986	(0.669, 1.454)	0.944
Low HDL cholesterol^c^										1.197	(0.807, 1.777)	0.371
Triglycerides ≥ 1.7 mmol/L										0.732	(0.466, 1.151)	0.177
Albuminuria										2.617	(1.725, 3.970)	< 0.001

*ACE* angiotensin-converting enzyme, *ARB* angiotensin II receptor antagonist, *CKD* chronic kidney disease, *CI* confidence interval, *HDL* high-density lipoprotein, *SD* standard deviation, *SGLT2* sodium glucose cotransporter 2

^a^Obesity defined as a body mass index ≥ 27 kg/m^2^,

^b^Median of duration of diabetes was 13 years

^c^Low HDL cholesterol defined as < 40 mg/dL (1.0 mmol/L) in men or < 50 mg/dL (1.3 mmol/L) in women

**Fig. 3:**
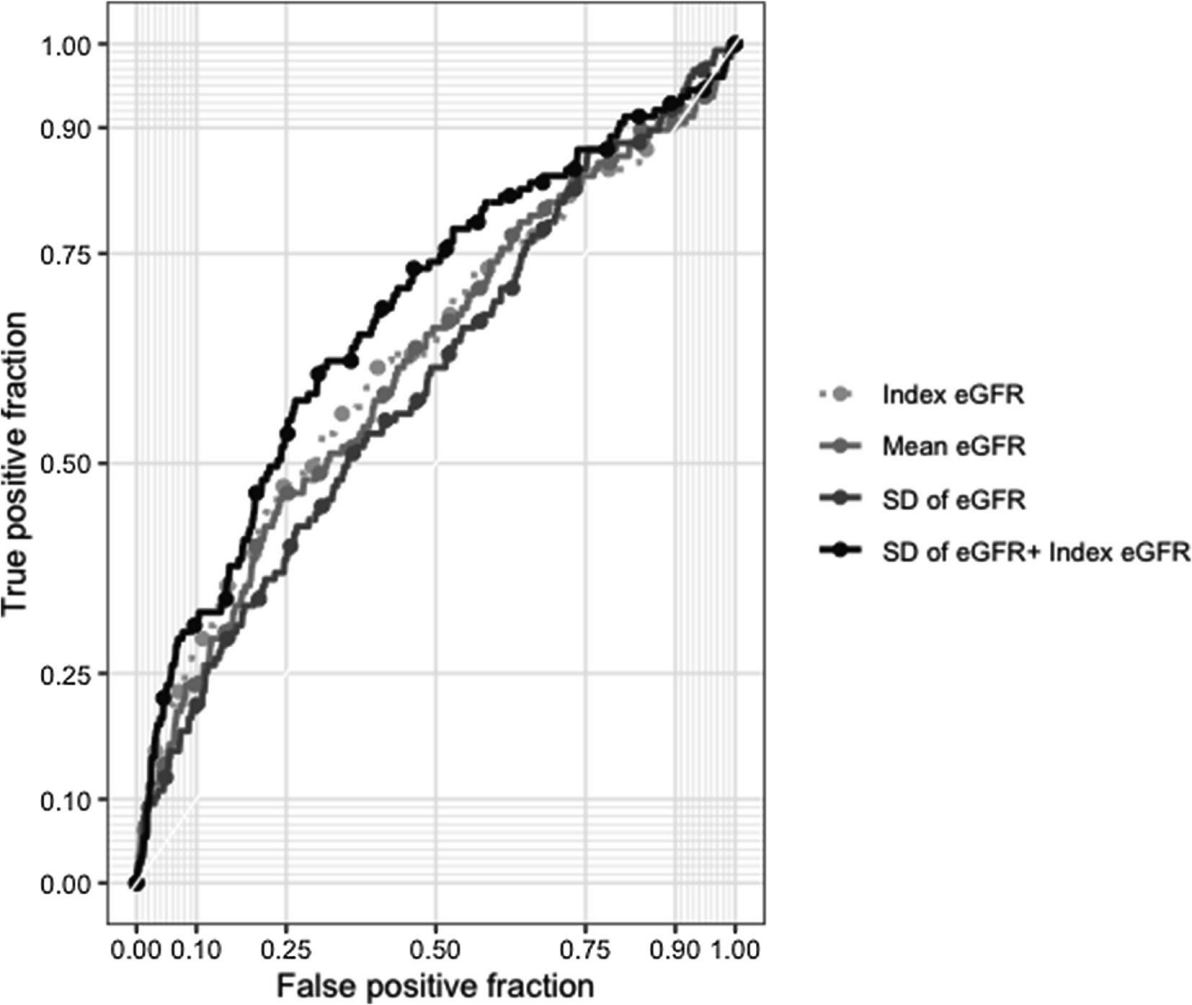
Receiver operating characteristic curves for the prediction of all-cause mortality in the index eGFR alone model, the mean of annual eGFR alone model, the SD of annual eGFR alone model, and the index eGFR + SD of annual eGFR model. *eGFR* estimated glomerular filtration rate, *SD* standard deviation

We generated the operating characteristic curves to differentiate all-cause mortality using the different models, including the index eGFR alone model, the mean of annual eGFR alone model, the SD of annual eGFR alone model, and the index eGFR + SD of annual eGFR model (Fig. 3). The C-index was significantly higher for the index eGFR + SD of annual eGFR model than for the index eGFR alone model [0.671 (95% CI 0.620‒0.723) vs. 0.629 (95% CI 0.574‒0.684), P < 0.001]. The C-index of the mean eGFR alone model [0.619 (95% CI 0.566‒0.673)] or that of the SD of annual eGFR alone model [0.593 (95% CI 0.541‒0.644)] was not significantly different compared with that of the index eGFR alone model (P = 0.846 and 0.800, respectively). To examine the superiority of the performance for the prediction of mortality using the index eGFR + SD of annual eGFR model to that using the index eGFR alone model, the IDI and NRI were calculated. The model of the index eGFR + SD of annual eGFR showed a significantly better IDI of 0.008 (95% CI 0.002‒0.023) and NRI of 0.141 (95% CI 0.017‒0.252) than the model of index eGFR alone (Table 3).

**Table 3:** The performance of the different models compared to the index eGFR model on the prediction of all-cause mortality

	C-index	P	IDI (95% CI)	P	NRI (95% CI)	P
Index eGFR	0.629 (0.574, 0.684)		Reference		Reference	
Mean eGFR	0.619 (0.566, 0.673)	0.846	− 0.003 (− 0.010, 0.002)	0.159	− 0.155 (− 0.336, 0.097)	0.246
SD of eGFR	0.593 (0.541, 0.644)	0.800	− 0.008 (− 0.022, 0.005)	0.199	− 0.154 (− 0.282, 0.011)	0.060
Index eGFR + SD of eGFR	0.671 (0.620, 0.723)	< 0.001	0.008 (0.002, 0.023)	0.007	0.141 (0.017, 0.252)	0.027

*CI*  confidence interval, *IDI*  integrated discrimination improvement, *NRI*  continuous net reclassification improvement, *eGFR*  estimated glomerular filtration rate, *SD*  standard deviation

**Fig. 4:**
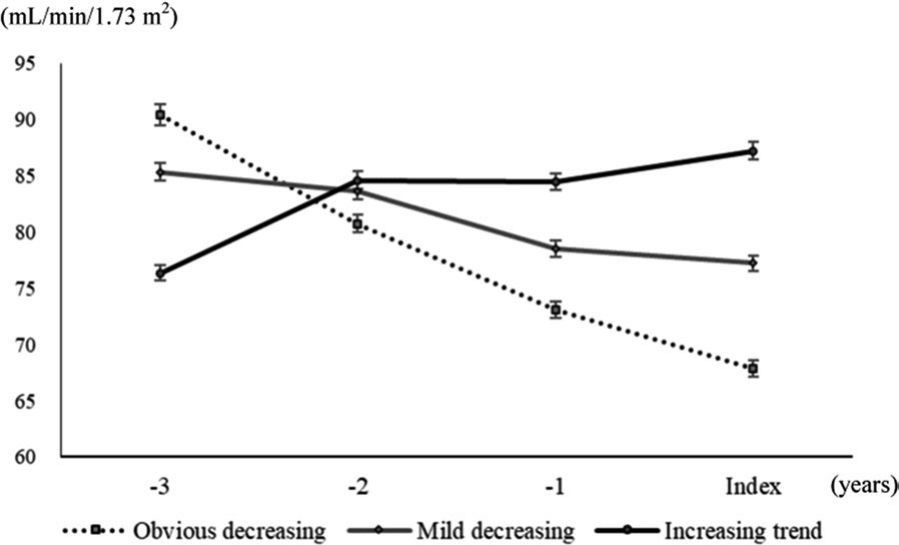
The trends of eGFR change over time among the three groups categorized based on the correlation between eGFR and time. Means and standard error bars of annual eGFR are plotted for each group. *eGFR* estimated glomerular filtration rate

To understand the relationship between the slopes of eGFR trajectories and mortality, we divided all patients into three groups according to the correlation between changes in eGFR and time. The patients with a positive rank coefficient of correlation were categorized as the increasing trend group (n = 1098), and the others were categorized into the mild decreasing group (n = 1247) and the obvious decreasing group (n = 1247) based on the median of the negative coefficients. The curves of eGFR changes over time are shown in Fig. 4. The incidences of mortality were 2.0, 2.3, and 2.4 deaths/100 person-years in the increasing trend group, the mild decreasing group, and the obvious decreasing group, respectively. There was no significant difference in the cumulative risk of mortality among these three groups assessed using Kaplan–Meier analysis (log-rank test P = 0.727).

## Discussion

The main finding in the present study is that previous variability in annual eGFR in the past 3 years had a synergistic effect with CKD for the prediction of all-cause mortality in patients with type 2 DM during a median follow-up of 19 months. Furthermore, variability in annual eGFR was an independent predictor of all-cause mortality in type 2 DM patients with an eGFR ≥ 60 mL/min/1.73 m^2^. In line with our findings, Al-Aly et al. [26] reported that previous variability in eGFR significantly predicted mortality in patients with an eGFR < 60 mL/min/1.73 m^2^. In patients with DM, Tseng et al. [21] reported that variability in eGFR significantly predicted the composite outcome of dialysis and mortality in patients with CKD, and Jun et al. [23] also reported that variability in eGFR significantly predicted the composite outcome but not mortality alone. The strength of our study is that we demonstrated that variability in eGFR has a synergistic effect with CKD on the prediction of the primary endpoint of all-cause mortality in patients with type 2 DM. In contrast to the above two studies, we retrospectively collected the SD of annual eGFR before the index eGFR. In clinical application, therefore, physicians can categorize the risk of patients when enrolled if they have previous annual eGFR data.

Moreover, in the present study, we found that an SD of annual eGFR ≥ the median of 7.62 mL/min/1.73 m^2^ could significantly predict all-cause mortality in patients with an eGFR ≥ 60 mL/min/1.73 m^2^. Although a low index eGFR seemed to predict mortality, CKD based on the index eGFR was no longer an independent predictor for mortality after adjusting for associated factors in patients with an SD of annual eGFR < 7.62 mL/min/1.73 m^2^. Hyperfiltration with a high eGFR was reported to be associated with increased mortality risk in patients with type 2 DM [17], and rapid change in eGFR might be a critical risk factor for mortality in those with a high eGFR [27]. Therefore, eGFR variability plays an important role in mortality risk and might be a better predictor than single-measured eGFR in type 2 DM patients with an eGFR ≥ 60 mL/min/1.73 m^2^.

There are several possible mechanisms underlying the association between high eGFR variability and increased mortality risk. First, variability in eGFR is associated with systemic inflammatory reactions. An episode of acute kidney injury, defined as highest serum creatinine > 150% or an increment ≥ 0.3 mg/dL compared to the lowest serum creatinine during hospitalization, was reported to predict major adverse outcomes and death in patients with type 2 DM independent of their CKD stage [28]. An increase in systemic inflammatory markers was observed in the renal ischemia–reperfusion model of mice [29]. Episodes of acute kidney injury, independent of renal failure, might induce leukocyte infiltration in distant organs, which was associated with cell apoptosis and dysfunction in the renal ischemic model of rats [30]. The data of annual eGFR assessed during hospitalization or in the emergency room in the past three years could have been collected, and the probable effect of an acute systemic disease on long-term mortality could not be excluded in the present study. Second, an increase in circulating glucose might induce renal hemodynamic changes and variability in eGFR [31]. Glucose variability might also induce endothelial dysfunction and result in micro- and macro-vascular diseases in patients with DM [32–35]. Therefore, the association between eGFR variability and increased mortality might result from unstable glucose control in patients with type 2 DM. Third, variability in eGFR might be reflected by active comorbidities, which are associated with long-term mortality [36]. The annual rate of eGFR decline was reported to be greater in patients with type 2 DM than in those without DM [37]. Insulin resistance might be associated with intrarenal hemodynamic dysfunction and predictive of all-cause mortality in patients with type 2 DM [38, 39]. Diabetic retinopathy was reported to be associated with eGFR variability and predictive of all-cause mortality in patients with type 2 DM [40, 41]. Peripheral artery disease (PAD) was also reported to be associated with CKD [42], and a high-risk PAD is predictive of all-cause mortality in patients with type 2 DM [43]. Moreover, Zhang et al. [44] verified that CKD was associated with a reduction in left ventricular function and strain based on cardiac magnetic resonance imaging in patients with type 2 DM. Variability in eGFR might be associated with left ventricular dysfunction, which is predictive of all-cause mortality [45].

Albuminuria is associated with a rapid eGFR decline in patients with type 2 DM [46, 47]. Moreover, UACR, rather than eGFR, is a significant predictor of mortality in patients with an eGFR ≥ 60 mL/min/1.73 m^2^ [7, 15]. On the other hand, an episode of acute kidney injury was also reported to significantly predict mortality after adjustment for albuminuria and CKD [28]. The prevalence of nonalbuminuric CKD has been increasing and is associated with long-term mortality in patients with DM [48, 49]. According to our findings in the present study, variability in eGFR, independent of albuminuria, could be a significant predictor of all-cause mortality in type 2 DM patients with preserved renal function.

In the present study, there was no significant difference in all-cause mortality risk among the three slope types of eGFR changes. Even in the subgroup of patients with a high SD of eGFR, the slope of eGFR change was not significantly associated with mortality risk (the data are not shown). In line with our results, both inclining and declining eGFR slopes were associated with a higher all-cause mortality risk than a stable eGFR level in patients with stage 3A CKD [50]. Furthermore, there was no significant difference in mortality prediction ability between the index eGFR and the mean eGFR based on the C-index, IDI, and NRI in the present study. Therefore, an episode of kidney injury might have a long-term effect on mortality risk [51, 52].

In the present study, the HbA1c level was significantly associated with CKD at enrollment, but an HbA1c ≥ 7% was not an independent predictor for all-cause mortality. High HbA1c levels have been reported to be associated with CKD in patients with type 2 DM [53, 54]. However, intensive glucose control did not decrease all-cause mortality in a median 10-years follow-up in the United Kingdom Prospective Diabetes Study (UKPDS) [55]. Although the risk of all-cause mortality was reduced during an additional 10 years of the UKPDS posttrial observation [56], a baseline HbA1c ≥ 7% could not significantly predict mortality during a median follow-up of 19 months in the present study. Notably, the use of SGLT2 inhibitors was an independent protector against mortality in the present study. It has been reported that SGLT2 inhibitors have a glucose-independent benefit on mortality reduction, and protection occurred early in the EMPA-REG outcome trial [57]. In line with our study, Schechter et al. [58] reported that the use of SGLT2 inhibitors reduced all-cause mortality based on real-world practice data. It has been reported that the benefits of SGLT2 inhibitors depend on body weight based on the medical datasets from a multicenter health care system in Taiwan [59]. Furthermore, there are sex-specific effects of DM on mortality [60]. The effect of structured personal diabetes care on the reduction of all-cause mortality risk was worse in men than in women [61]. Consistently, male sex was an independent predictor for all-cause mortality in the present study.

There are several potential limitations that should be acknowledged in the present study. First, we collected only annual eGFR data instead of all available eGFR data. The advantage of using annual eGFR is that it is compatible with clinical practice, and the similar interval between the four eGFR data points prevents the effects of frequent measurements on SD. However, some episodes of short-term eGFR alteration might have been missed in the present study. Second, we used the MDRD equation for eGFR because the data were automatically calculated in our hospital information system. The Cockcroft and Gault (C–G) equation was reported to be more convincing for medicine-dose adjustment in the aged or female population [62]. However, the measurement of serum creatinine levels might occur several days earlier than the body-weight measurement for an outpatient interview, and we did not use the C–G equation to avoid this time-lag induced bias. The MDRD equation has been reported to have the highest accuracy for measuring eGFR based on 24-h urine collection compared with the C–G equation and the Chronic Kidney Disease Epidemiology Collaboration (CKD-EPI) equation among the population with DM [63]. Although the CKD-EPI equation has been reported to be better in mortality prediction than the MDRD equation in patients with type 2 DM [64], there is no consensus on the use of the CKD-EPI equations which should be modified for application in Chinese population [65]. Furthermore, the use of the CKD-EPI equation is still not validated in eGFR variability, which might be increased due to the use of different formulae if serum creatinine varies across 0.7 mg/dL in females and 0.9 mg/dL in males [65]. Third, we did not exclude patients who began to use ACE inhibitors, ARBs, or SGLT2 inhibitors during the period of annual eGFR data collection. These medications may cause transient reduction in eGFR values. Fourth, we did not directly investigate the mechanisms or potential comorbidities associated with eGFR variability and mortality. Finally, this was an observational study that used retrospectively collected annual eGFR data. We did not investigate the effect of any specific intervention on eGFR variability and mortality. Our results cannot indicate that mortality will be reduced by decreasing eGFR variability.

## Conclusion

In the present study, we found a synergistic effect of CKD and previous eGFR variability on all-cause mortality in patients with type 2 DM. In addition, the SD of annual eGFR might be an independent predictor of mortality in patients with preserved renal function (eGFR ≥ 60 mL/min/1.73 m^2^), but single-measured eGFR was not a significant predictor of mortality in patients with low eGFR variability after adjustment for the associated factors. This observational cohort study indicates a possible linkage between eGFR variability and long-term mortality; further large-scale studies regarding interventions to reduce eGFR variability to prevent mortality are warranted.

## Supplementary Information

**Additional file 1: Table S1.** The baseline characteristics and mortality cause of expired patients categorized based on CKD and SD of eGFR.

## Data Availability

The datasets used and/or analyzed during the current study are available from the corresponding author on reasonable request.

## Abbreviations

ACE: Angiotensin-converting enzyme
ARB: Angiotensin II receptor antagonists
BMI: Body mass index
C–G: Cockcroft and Gault
CKD: Chronic kidney disease
CKD-EPI: Chronic Kidney Disease Epidemiology Collaboration
CI: Confidence interval
CVD: Cardiovascular disease
DM: Diabetes mellitus
eGFR: Estimated glomerular filtration rate
HbA1c: Hemoglobin A1c
HDL: High-density lipoprotein
HR: Hazard ratio
IDI: Integrated discrimination improvement
NRI: Net reclassification improvement
PAD: Peripheral artery disease
SD: Standard deviation
SGLT2: Sodium glucose cotransporter 2
UACR: Urinary albumin-to-creatinine ratio
UKPDS: United Kingdom Prospective Diabetes Study
