# Supplementary material for: A synergistic effect of variability in estimated glomerular filtration rate with chronic kidney disease on all-cause mortality prediction in patients with type 2 diabetes: a retrospective cohort study
Source: Cardiovasc Diabetol. 2021 Oct 18;20:209. doi: 10.1186/s12933-021-01399-z (PMC8524871; doi:10.1186/s12933-021-01399-z)
Supplement: Supplementary file 1 — Additional file 1: Table S1. The baseline characteristics and mortality cause of expired patients categorized based on CKD and SD of eGFR. [file 12933_2021_1399_MOESM1_ESM.docx]

Additional file 1: Table S1. The baseline characteristics and mortality cause of expired patients categorized based on CKD and SD of eGFR

|  | Low SD without CKD  (n = 22) | | High SD without CKD  (n = 44) | | Low SD with CKD  (n = 27) | | High SD with CKD  (n = 34) | | P |
| --- | --- | --- | --- | --- | --- | --- | --- | --- | --- |
| Age (year) | 76.8 | ± 11.5 | 70.3 | ± 10.9 | 78.5 | ± 10.6 | 75.8 | ± 10.3 | 0.010 |
| Male, n (%) | 15 | (68.2%) | 28 | (63.6%) | 17 | (63.0%) | 20 | (58.8%) | 0.915 |
| Current smoking, n (%) | 3 | (13.6%) | 4 | (9.1%) | 0 | (0.0%) | 3 | (8.8%) | 0.328 |
| CVD history, n (%) | 7 | (31.8%) | 6 | (13.6%) | 11 | (40.7%) | 12 | (35.3%) | 0.053 |
| BMI (kg/m^2^) | 24.3 | ± 4.9 | 24.9 | ± 4.1 | 27.3 | ± 5.5 | 25.3 | ± 5.4 | 0.161 |
| Duration of diabetes (year) | 16.7 | ± 7.1 | 14.9 | ± 8.0 | 18.7 | ± 11.1 | 17.4 | ± 10.9 | 0.306 |
| Hypertension, n (%) | 19 | (86.4%) | 40 | (90.9%) | 27 | (100.0%) | 32 | (94.1%) | 0.280 |
| Systolic BP (mmHg) | 139 | ± 28 | 136 | ± 23 | 148 | ± 20 | 143 | ± 26 | 0.091 |
| Diastolic BP (mmHg) | 76 | ± 11 | 75 | ± 14 | 76 | ± 13 | 72 | ± 13 | 0.686 |
| HbA1c (%) | 7.8 | ± 1.7 | 7.3 | ± 1.5 | 7.6 | ± 2.0 | 7.8 | ± 1.9 | 0.754 |
| Total cholesterol (mmol/L) | 4.2 | ± 1.2 | 4.1 | ± 1.3 | 3.9 | ± 0.8 | 3.6 | ± 0.7 | 0.245 |
| HDL cholesterol (mmol/L) | 1.3 | ± 0.4 | 1.3 | ± 0.4 | 1.2 | ± 0.5 | 1.2 | ± 0.4 | 0.927 |
| Triglycerides (mmol/L) | 1.4 | ± 1.2 | 1.2 | ± 0.6 | 1.6 | ± 0.7 | 1.5 | ± 0.9 | 0.022 |
| Index eGFR (mL/min/1.73 m^2^) | 87.9 | ± 22.5 | 90.5 | ± 21.8 | 40.9 | ± 13.2 | 34.7 | ± 14.0 | <0.001 |
| Mean of eGFR (mL/min/1.73 m^2^) | 88.2 | ± 22.4 | 93.3 | ± 23.6 | 42.7 | ± 11.8 | 50.8 | ± 18.3 | <0.001 |
| SD of eGFR (mL/min/1.73 m^2^) | 5.6 | ± 1.5 | 18.0 | ± 8.9 | 4.5 | ± 1.5 | 15.1 | ± 11.3 | <0.001 |
| Albuminuria | 2 | (9.1%) | 9 | (20.5%) | 13 | (48.1%) | 17 | (50.0%) | 0.001 |
| ACE inhibitor or ARB, n (%) | 12 | (54.5%) | 18 | (40.9%) | 18 | (66.7%) | 21 | (61.8%) | 0.132 |
| Antiplatelet, n (%) | 10 | (45.5%) | 14 | (31.8%) | 16 | (59.3%) | 20 | (58.8%) | 0.055 |
| Statins, n (%) | 14 | (63.6%) | 25 | (56.8%) | 17 | (63.0%) | 20 | (58.8%) | 0.935 |
| Insulin therapy, n (%) | 7 | (31.8%) | 14 | (31.8%) | 4 | (14.8%) | 20 | (58.8%) | 0.004 |
| Oral antidiabetic drugs | 18 | (81.8%) | 33 | (75.0%) | 23 | (85.2%) | 29 | (85.3%) | 0.624 |
| Insulin secretagogues, n (%) | 6 | (27.3%) | 15 | (34.1%) | 14 | (51.9%) | 13 | (38.2%) |  |
| Metformin, n (%) | 9 | (40.9%) | 10 | (22.7%) | 5 | (18.5%) | 4 | (11.8%) |  |
| Thiazolidinediones, n (%) | 6 | (27.3%) | 7 | (15.9%) | 9 | (33.3%) | 2 | (5.9%) |  |
| DPP4 inhibitors, n (%) | 9 | (40.9%) | 24 | (54.5%) | 21 | (77.8%) | 23 | (67.6%) |  |
| SGLT2 inhibitors, n (%) | 1 | (4.5%) | 0 | (0.0%) | 0 | (0.0%) | 1 | (2.9%) |  |
| Causes of mortality |  |  |  |  |  |  |  |  | 0.005 |
| Malignancy, n (%) | 8 | (36.4%) | 29 | (66.0%) | 5 | (18.5%) | 11 | (32.4%) |  |
| CVD, n (%) | 7 | (31.8%) | 7 | (15.9%) | 9 | (33.4%) | 15 | (44.1%) |  |
| Infection, n (%) | 2 | (9.1%) | 2 | (4.5%) | 5 | (18.5%) | 1 | (2.9%) |  |
| Others, n (%) | 5 | (22.7%) | 6 | (13.6%) | 8 | (29.6%) | 7 | (20.6%) |  |

Continuous data are presented as the mean ± SD, and the Kruskal-Wallis test was conducted to detect significant differences among the groups.

Categorical data are presented as numbers (percentages), and the chi-square test was conducted to detect differences among the groups.

CKD was defined as an index eGFR < 60 mL/min/1.73 m^2^, and SD of eGFR was grouped based on the median of 7.62 mL/min/1.73 m^2^.

ACE = angiotensin-converting enzyme, ARB = angiotensin II receptor antagonist, BMI = body mass index, BP = blood pressure, CVD = cardiovascular disease, DPP4 = dipeptidyl peptidase-4, eGFR = estimated glomerular filtration rate, HbA1c = hemoglobin A1c, HDL = high-density lipoprotein, SD = standard deviation, SGLT2 = sodium glucose cotransporter 2.
